# Enhancer RNA-driven looping enhances the transcription of the long noncoding RNA DHRS4-AS1, a controller of the *DHRS4* gene cluster

**DOI:** 10.1038/srep20961

**Published:** 2016-02-11

**Authors:** Yingying Yang, Zhongjing Su, Xuhong Song, Bin Liang, Fanxing Zeng, Xiaolan Chang, Dongyang Huang

**Affiliations:** 1 Department of Cell Biology and Genetics and Key Laboratory of Molecular Biology in High Cancer Incidence Coastal Chaoshan Area of Guangdong Higher Education Institutes, Shantou University Medical College, Shantou 515041, China

## Abstract

The human *DHRS4* gene cluster consists of *DHRS4* and two immediately downstream homologous genes, *DHRS4L2* and *DHRS4L1*, generated by evolutionarily gene-duplication events. We previously demonstrated that a head-to-head natural antisense transcript (NAT) of *DHRS4*, denoted *DHRS4-AS1*, regulates all three genes of the *DHRS4* gene cluster. However, it is puzzling that *DHRS4L2* and *DHRS4L1* did not evolve their own specific NATs to regulate themselves, as it seems both have retained sequences highly homologous to *DHRS4-AS1*. In a search of the *DHRS4-AS1* region for nearby enhancers, we identified an enhancer located 13.8 kb downstream of the *DHRS4-AS1* transcriptional start site. We further showed, by using a chromosome conformation capture (3C) assay, that this enhancer is capable of physically interacting with the *DHRS4-AS1* promoter through chromosomal looping. The enhancer produced an eRNA, termed AS1eRNA, that enhanced *DHRS4-AS1* transcription by mediating the spatial interactions of the enhancer and *DHRS4-AS1* promoter in cooperation with RNA polymerase II and p300/CBP. Moreover, the distributions of activating acetyl-H3 and H3K4me3 modifications were found to be greater at the *DHRS4-AS1* promoter than at the homologous duplicated regions. We propose that AS1eRNA-driven DNA looping and activating histone modifications promote the expression of *DHRS4-AS1* to economically control the *DHRS4* gene cluster.

The human *DHRS4* gene, located on chromosome 14q11.2, encodes an NADPH-dependent enzyme (NRDR) belonging to the short-chain dehydrogenase/reductase (SDR) family^1^,^2^. Human *DHRS4* forms a gene cluster (Fig. 1A) with its two immediately downstream homologous genes, *DHRS4L2* and *DHRS4L1*, generated by recent gene-duplication events^3^. *DHRS4* is highly conserved among mammals and many other species, whereas *DHRS4L1* and *DHRS4L2* are present only in primates. Phylogenetic analysis of the *DHRS4* gene cluster suggests that *DHRS4L2* and *DHRS4L1* are paralogues of *DHRS4*, and that *DHRS4L2* is the newest member^4^.

Natural antisense transcripts (NATs) are long non-coding (lnc) RNA molecules transcribed from the antisense strand of protein-coding and non-protein-coding genes, and there are three types: head-to-head, tail-to-tail, and fully overlapping relative to the respective sense transcripts^5^. We previously identified the head-to-head NAT *DHRS4-AS1* and demonstrated its role in silencing of all three genes of the *DHRS4* gene cluster by recruitment of epigenetic modifiers^6^. The transcriptional start site (TSS) of *DHRS4-AS1* is located in the first intron of *DHRS4* (Fig. 1A). Because the *DHRS4* gene underwent duplication to produce *DHRS4L2* and *DHRS4L1* in tandem, the duplicated exon1, exon2 and promoter of *DHRS4-AS1* are retained at the corresponding sites within *DHRS4L2* and *DHRS4L1*. However, local head-to-head NATs from *DHRS4L2* and *DHRS4L1* are not found, but for convenience we named the putative NATs as *DHRS4L2-AS* and *DHRS4L1-AS*. *DHRS4-AS1* is a specific product of primate evolution. Mice, for example, have a transcribed head-to-head 834-bp single-exon NAT for *Dhrs4*, but the locus has a different gene configuration and a different pattern of regulation^6^.

As DNA regulatory elements, enhancers can control the activation of their target genes and play a central role in mediating long-range chromatin interactions to enable target-gene expression^7^,^8^,^9^. Enhancer-derived RNAs (eRNAs) are a class of lncRNAs synthesized from active enhancers marked by histone H3 lysine 4 monomethylation (H3K4me1), histone H3 lysine 27 acetylation (H3K27ac) and presence of RNA polymerase II (RNA pol II)^10^,^11^. eRNAs can be bidirectional non-polyadenylated or unidirectional polyadenylated transcripts, and their levels can correlate with those of their target gene^12^. It is still unclear whether eRNAs are merely by-products of enhancer activation or whether they might serve as key regulators of gene expression^8^,^11^,^13^. However, recent evidence has shown that some eRNAs can increase the expression of target genes by mediating enhancer–promoter looping or facilitating RNA pol II loading^14^,^15^,^16^.

Here, we show that a newly identified enhancer located 13.8 kb downstream of the *DHRS4-AS1* TSS can produce an eRNA (AS1eRNA) that enhances only *DHRS4-AS1* expression, not the local antisense transcription for *DHRS4L2* and *DHRS4L1*. In cooperation with RNA pol II and p300 histone acetyltransferase, AS1eRNA enhances transcription of *DHRS4-AS1* by mediating chromatin looping between the enhancer and the *DHRS4-AS1* gene promoter.

## Results

### Identification of the downstream enhancer region of *DHRS4-AS1*

**Figure 1:**
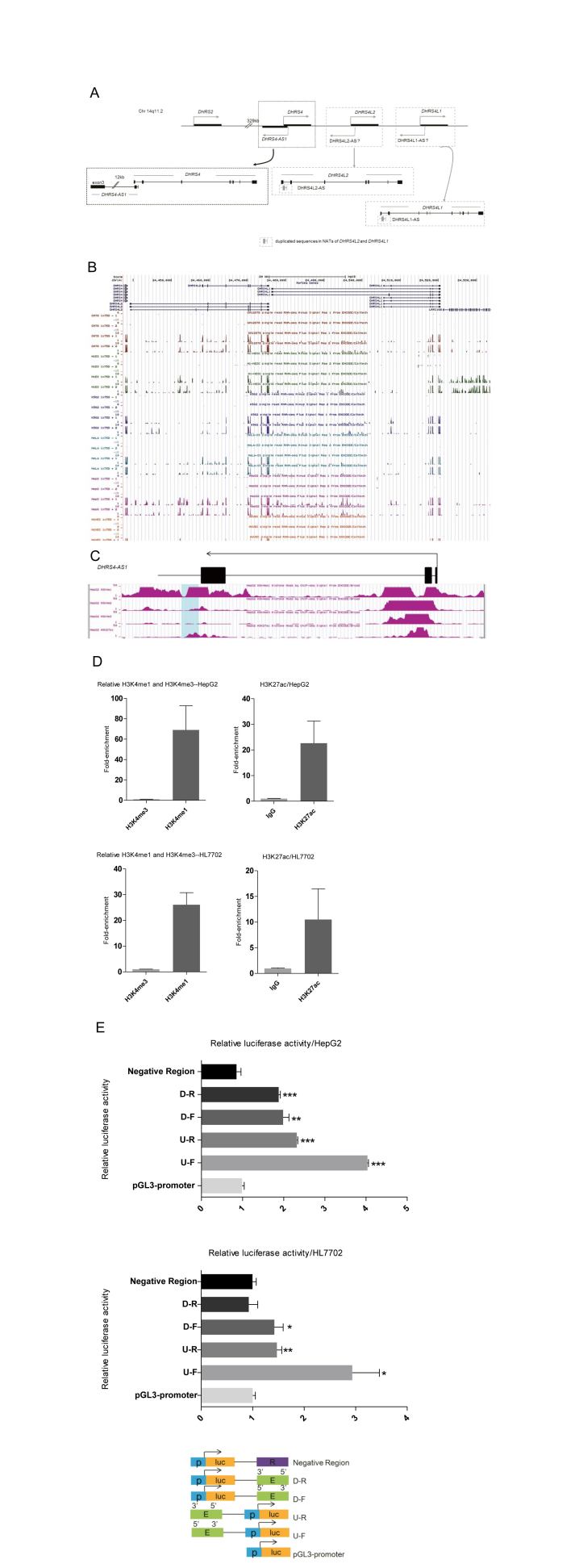
A predicted downstream enhancer region of *DHRS4-AS1* shows enhancer activity. (**A**) Structure of the human *DHRS4* gene cluster and corresponding putative NATs at chromosomal band 14q11.2. In the gene duplication events that gave rise to *DHRS4L2* and *DHRS4L1* from *DHRS4*, at least some of the sequences of *DHRS4-AS1* present in *DHRS4* could, in principle, be retained in *DHRS4L2* and *DHRS4L1*. The gray box (bottom) represents the duplicated sequences of *DHRS4-AS1* that were retained in *DHRS4L2* and *DHRS4L1*. (**B**) ENCODE tracks from the UCSC Genome Browser using strand-specific RNA-seq failed to identify evidence of transcription of *DHRS4L2-AS* and *DHRS4L1-AS* from the minus strand in GM12818, H1-hESC, K562, HeLa-S3, HepG2 and HUVEC cells. (**C**) Localization of the putative enhancer element at the 3′ end of the *DHRS4-AS1* gene. ENCODE tracks from the UCSC Genome Browser illustrate the high levels of H3K4me1 and H3K27ac, and the low levels of H3K4me3 in HepG2 cells. The light blue rectangle represents the predicted enhancer region. (**D**) HepG2 and HL7702 cells were subjected to ChIP for verification of H3K4me1, H3K4me3 and H3K27ac. The abundance of histone modifications within the predicted enhancer region is in accordance with the ENCODE tracks of histone modifications from the UCSC Genome Browser database. (**E**) The downstream predicted enhancer elevates promoter-driven luciferase activity. The four constructs [upstream/forward (U-F), upstream/reverse (U-R), downstream/forward (D–F) and downstream/reverse (D–R)] were co-transfected with a Renilla reporter gene into HepG2 and HL7702 cells. Random DNA (R) represents the negative control harboring no enhancer features. Luciferase activity was normalized to Renilla luciferase activity and then divided by the values for the pGL3 promoter empty vector control. The positions and orientations of the constructs are shown (lower panel). All data shown are the mean ± standard error of the mean (SEM) of at least three independent experiments. *P* values were determined by Student’s unpaired two-tailed t test. *P < 0.05, **P < 0.01, ***P < 0.001.

The human *DHRS4* gene cluster contains *DHRS4* and its two orthologous duplications *DHRS4L2* and *DHRS4L1*. The mRNA sequences of *DHRS4L2* and *DHRS4L1* share high homology with that of *DHRS4* (>80%), and the DNA sequence homology of all three genes exceeds 77%. The TSS of *DHRS4-AS1* is within the first intron of *DHRS4* (Fig. 1A). Predicted promoters for putative NATs can be found within the antisense strands of *DHRS4L2* and *DHRS4L1*, and the levels of DNA homology of the predicted promoter regions exceed 90% (see Supplementary Table S1). However, we failed to find evidence of transcription of *DHRS4L2-AS* and *DHRS4L1-AS*, and this absence is also reflected in the strand-specific RNA-seq database of the UCSC ENCODE project (Fig. 1B). Features were visualized in the UCSC Genome Browser (http://genome.ucsc.edu)^17^,^18^.

Active enhancers are marked by high levels of H3K4me1, low levels of H3K4me3, and high levels of H3K27ac^19^,^20^,^21^. Based on the histone modifications catalogued in the UCSC Genome Browser (Fig. 1C)^17^,^18^, we searched for and identified a putative enhancer region located 13.8 kb downstream from the TSS of *DHRS4-AS1*^17^,^18^. We performed chromatin immunoprecipitation (ChIP) to confirm the enrichment of histone modifications in hepatocellular carcinoma cells (HepG2) and normal hepatocytes (HL7702) (Fig. 1D). We then examined the potential enhancement of luciferase reporter activity by using luciferase reporter assays in HepG2 and HL7702 cells (Fig. 1E). We isolated a 1.6-kb putative enhancer region (termed the AS1 enhancer) and cloned it upstream of the promoter of the pGL3 luciferase reporter vector while the downstream insertion was inserted to 3′ to the luciferase reporter and SV40 late polyadenylation signal in both forward and reverse orientations (see Fig. 1E). Transfection of all reporters revealed approximately 2- to 4-fold enhancement of luciferase reporter activity, whereas a genomic region harboring no enhancer features (i.e. a control) did not show any activity in HepG2 cells (Fig. 1E). As expected of an enhancer region, enhancement of luciferase reporter activity by the AS1 enhancer was orientation- and position- independent. We observed weaker enhancement of luciferase reporter activity by the enhancer in HL7702 cells compared to HepG2 cells. Altogether, we identified a predicted AS1 enhancer region that harbors features of enhancer domains and possesses enhancer activity.

### The AS1 enhancer selectively mediates long-range interactions with the *DHRS4-AS1* promoter

**Figure 2:**
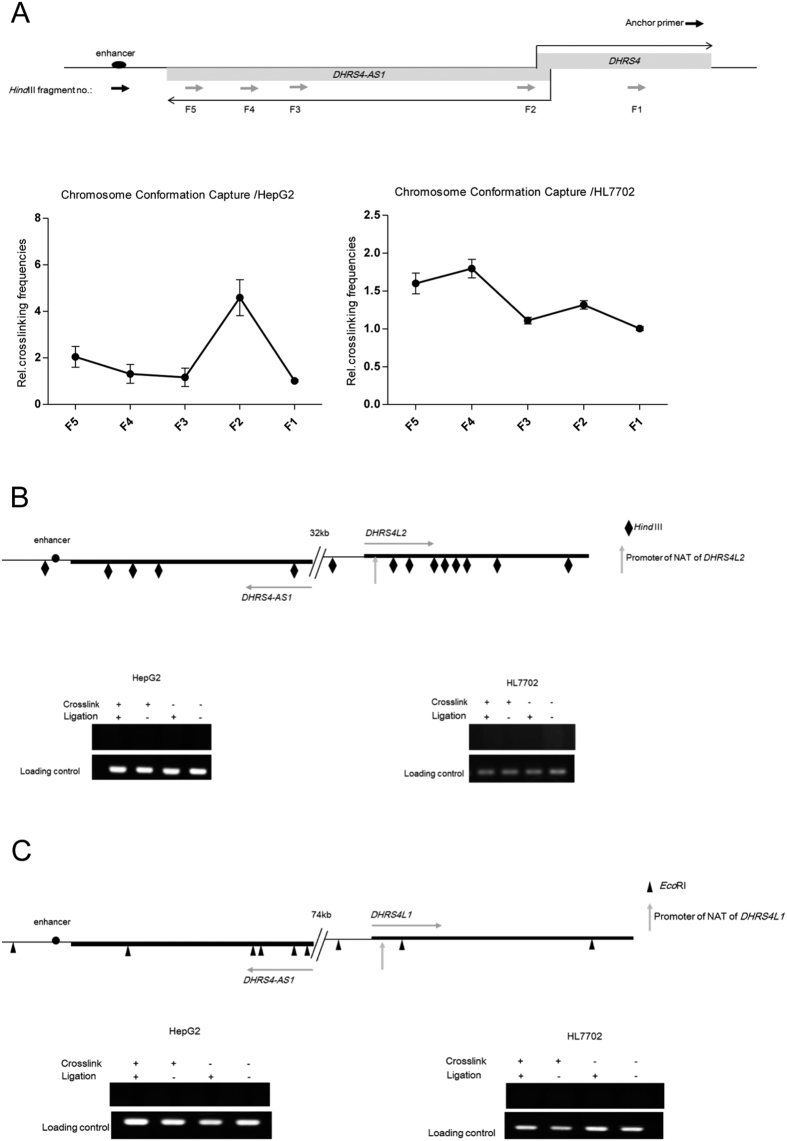
AS1 enhancer selectively interacts with *DHRS4-AS1* promoter. Cells were crosslinked with 1% formaldehyde, and then the reaction was stopped by the addition of glycine. Restriction enzymes *Hind*III or *Eco*RI were used to digest the crosslinked chromatin. Then ligation was performed by incubation with T4 DNA ligase. Purified ligation products were determined by qPCR. (**A**) The relative crosslinking frequencies between the anchor region (the AS1 enhancer) and distal fragments (F1~F5) were measured by qPCR and normalized to the control region (fragment F1). Error bars indicate the mean ± SEM of three experiments. *P* values were determined by Student’s unpaired two-tailed t test. (**B**,**C**) Spatial interactions between the AS1 enhancer and homologous promoter regions of putative *DHRS4L2* and *DHRS4L1* NATs were determined by 3C array. Crosslinked chromatin was then digested with *Hind*III (**B**) or *Eco*RI (**C**), followed by ligation. 3C samples from *Hind*III- (**B**) or *Eco*RI- (**C**) digested crosslinked chromatin without ligation and non-crosslinked genomic DNA with or without ligation were used as negative controls. To ensure the various primer pairs all worked as intended, we performed a random ligation control where PCR was performed to amplify DNA from BAC clones. The primers used here effectively amplified the control templates that contained all the ligation products. Notably, the putative promoter sequences used in this analysis contain all the potential promoters predicted from PROSCAN (see Methods) at the putative homologous NATs of *DHRS4L2* and *DHRS4L1*. The PCR products from 3C samples, not cut with any restriction enzyme, were used as the loading control.

Numerous enhancers gain close proximity to their target promoters by forming DNA loops^22^. To further explore whether the AS1 enhancer element is able to interact from a distance with the *DHRS4-AS1* promoter, we performed chromosome conformation capture (3C) analysis to quantitatively measure chromosomal interactions between the AS1 enhancer and the *DHRS4-AS1* promoter (Fig. 2A). To identify whether there is a chromatin loop between two segments, it needs to be demonstrated that the two segments interact more frequently with each other than with neighboring DNA fragments^23^. In HepG2 cells, we found that the *DHRS4-AS1* promoter (represented by fragment F2 in Fig. 2A) was the most prominent genomic locus to interact with the AS1 enhancer (*P* < 0.05). This was not as evident in HL7702 cells compared to in HepG2 cells, but statistical analysis still enabled the conclusion that there is a chromatin loop between the AS1 enhancer and the *DHRS4-AS1* promoter. In HL7702 cells, the enhancer shows significantly more frequent interaction with F2 than its neighboring DNA fragments F1 and F3 (*P* < 0.05), and the error bars of F1/F3 sites do not overlap with the error bars of the F2/F4 sites.

To assess if the AS1 enhancer selectively interacts with the *DHRS4-AS1* promoter within the *DHRS4* gene cluster, we also characterized interactions of the AS1 enhancer and promoter regions within the putative homologous NATs of *DHRS4L2* and *DHRS4L1* by performing chromosome conformation capture (Fig. 2B,C). *Hind*III and *Eco*RI were used to digest nuclei to determine the interactions between the AS1 enhancer and the putative *DHRS4L2* and *DHRS4L1* NAT promoters. Physical interactions were determined by PCR primers that amplify the ligated hybrid fragments containing the AS1 enhancer and the putative *DHRS4L2* or *DHRS4L1* NAT promoters. *Hind*III- (Fig. 2B) or *Eco*RI- (Fig. 2C) digested crosslinked chromatin without ligation and non-crosslinked genomic DNA with or without ligation were used as negative controls. A random ligation control where PCR was performed was used to amplify DNA from BAC clones to ensure the various primer pairs all worked as intended. The primers used here effectively amplified the control templates that contained all the ligation products. The PCR results revealed that there were no interactions between the AS1 enhancer and the homologous NAT promoters for *DHRS4L2* and *DHRS4L1*.

### AS1eRNA-driven DNA looping enhances *DHRS4-AS1* gene expression

**Figure 3:**
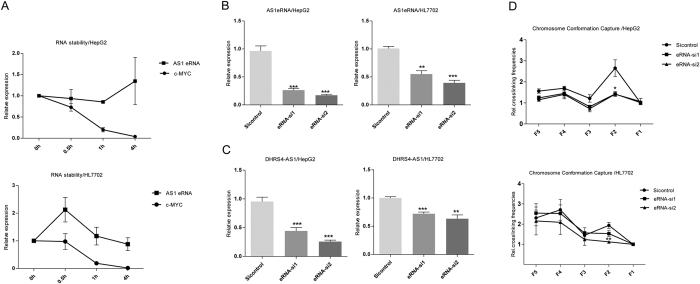
AS1eRNA enhances *DHRS4-AS1* transcription by mediating DNA looping between the *DHRS4-AS1* promoter and its enhancer. (**A**) AS1eRNA is stable following treatment with actinomycin D. Cells were treated with 5 μg/ml actinomycin D, using c-MYC as the positive control in HepG2 and HL7702 cells. (**B,C**) After siAS1eRNA treatment, qPCR analysis of AS1eRNA and DHRS4-AS1 levels was performed at 48 h. The analysis of RNA levels used β-actin as the internal control. The siRNA-control was a scrambled sequence with no homology to any known gene. (**D**) The relative crosslinking frequencies of the AS1 enhancer/*DHRS4-AS1* promoter were determined by qPCR. We first normalized the bias of the PCR efficiency among each primer set we used. This was performed by measuring the corresponding amplifications from a bacterial artificial chromosome, which was digested with *Hind*III and ligated with T4 DNA ligase. For correcting the differences of crosslinking and digestion efficiencies between samples, *ERCC3* “control interaction frequencies” were used as internal control (see Methods). The relative crosslinking frequencies of F1~F5 was determined the fold change relative to the control region (F1). *P* values were determined by Student’s unpaired two-tailed t test. Error bars represent the mean ± SEM of three independent experiments. *P < 0.05, **P < 0.01, ***P < 0.001.

Recent evidence indicates that eRNAs can interact with looping factors to facilitate chromosomal looping between the enhancer and the promoter(s) of target gene(s)^24^. By performing 5′ and 3′ rapid amplification of cDNA ends (RACE) based on the known AS1 enhancer region, we cloned a 655-bp transcript, designated AS1eRNA, which was transcribed from the antisense strand and consists of only one exon with a 3′-polyadenylated tail (see Supplementary Fig. S1) [GenBank: KT282098]. By using the Coding Potential Calculator, which annotates human lnc genes, we classified AS1eRNA as a non-coding RNA (see Supplementary Fig. S1)^25^. Most eRNAs are less stable bidirectional transcripts^26^,^27^. Whether unidirectional, polyA-containing AS1eRNA is less stable is unknown. We therefore investigated the stability of AS1eRNA by using actinomycin D to inhibit total cellular transcription in HepG2 and HL7702 cells, using c-MYC as a control for a short half-life mRNA species (Fig. 3A)^28^. The degradation of c-MYC was rapid following treatment with actinomycin D, between 30 min and 4 h, but AS1eRNA was stable.

To determine whether AS1eRNA plays an important role in regulating *DHRS4-AS1*, two short interfering RNAs (siRNAs) that specifically target the exon region of AS1eRNA (siRNA-1, siRNA-2) were designed. Both siRNAs caused a significant reduction of eRNA. The inhibition by siRNA-1 on AS1eRNA expression was as much as 74% in HepG2 and 45% in HL7702 cells, and the inhibition by siRNA-2 was 82% in HepG2 and 60% in HL7702 cells (Fig. 3B). Knockdown of AS1eRNA led to a significant decrease in *DHRS4-AS1* expression in both HepG2 and HL7702 cells compared to cells treated with the non-targeting control siRNA, with a greater inhibitory effect in HepG2 cells than in HL7702 cells (Fig. 3C). RT-qPCR results showed that siRNA-induced depletion of AS1eRNA in HepG2 cells led to a reduction in the level of *DHRS4-AS1* transcript by 55% using siRNA-1 and by 74% using siRNA-2; in HL7702 cells, the level of *DHRS4-AS1* transcript was reduced by 28% using siRNA-1 and by 36% using siRNA-2.

To further investigate the potential role of AS1eRNA on *DHRS4-AS1* transcription, we treated cells with siRNAs and then performed 3C-qPCR to assess the interaction of the AS1 enhancer and the *DHRS4-AS1* promoter. AS1eRNA knockdown by siRNA-1 and siRNA-2 resulted in a reduction of the cross-linking frequencies in HepG2 cells, but in HL7702 cells, we just observed the effect of siRNA-2 on the interaction (Fig. 3D). Collectively, these results suggest that AS1eRNA enhances *DHRS4-AS1* transcription by forming DNA loops.

### AS1eRNA mediates long-range chromatin interactions with RNA pol II and p300 between the AS1 enhancer and the *DHRS4-AS1* promoter

**Figure 4:**
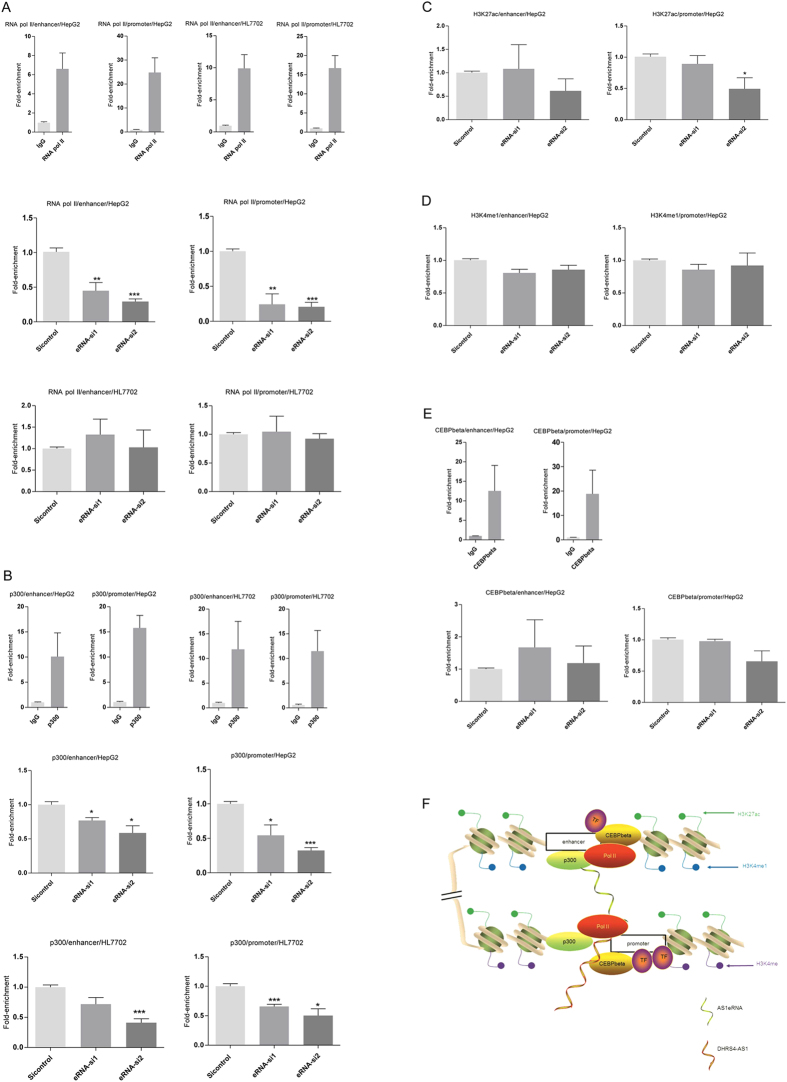
RNA pol II and p300 mediate spatial interactions between the *DHRS4-AS1* promoter and its enhancer. (**A**,**B**) HepG2 and HL7702 cells were subjected to ChIP to detect changes in enrichment of RNA pol II and p300 at the AS1 enhancer and the *DHRS4-AS1* promoter after siAS1eRNA treatment. (**C–E**) ChIP analysis was performed to investigate the changes in levels of histone modifications H3K27ac and H3K4me1, and transcription factor CEBPbeta in HepG2 cells. Data are shown as fold-enrichment relative to the siRNA-control. *P* values were determined by Student’s unpaired two-tailed t test. Error bars represent the mean ± SEM of three independent experiments. *P < 0.05, **P < 0.01, ***P < 0.001. (**F**) A working model shows the possible role of AS1eRNA on *DHRS4-AS1*. The enrichment of RNA pol II, p300, H3K4me1, H3K27ac and CEBPbeta establishes open chromatin to produce AS1eRNA. In cooperation with RNA pol II and p300, the AS1 enhancer spatially interacts with the *DHRS4-AS1* promoter in an eRNA-dependent manner. The interactions enhance the transcription of *DHRS4-AS1*. After knockdown of AS1eRNA, the distributions of RNA pol II and p300 at the AS1 enhancer and *DHRS4-AS1* promoter are reduced, and result in a reduction of *DHRS4-AS1* levels.

RNA pol II and the transcriptional coactivator p300/CBP occupy enhancer regions and target gene promoters to maintain chromatin loops^29^. We next characterized the effect of AS1eRNA knockdown on the binding levels of RNA pol II and p300 at the AS1 enhancer and the *DHRS4-AS1* promoter (Fig. 4A,B). Depletion of AS1eRNA resulted in reduced RNA pol II and p300 binding to both the AS1 enhancer and the *DHRS4-AS1* promoter in HepG2 cells. However, in HL7702 cells, depletion of AS1eRNA did not induce a significant change in RNA pol II binding, although AS1eRNA knockdown by siRNA-2 did inhibit the enrichment of p300 at both the AS1 enhancer and the *DHRS4-AS1* promoter, which may owe to the relatively weak AS1 enhancer activity in this cell line, inferred from the dual-luciferase reporter assays and 3C assays. As a transcriptional factor, previous reports have shown that p300 can interact with CEBPbeta as a binding partner^30^,^31^. In addition, enrichment of CEBPbeta at the AS1 enhancer and *DHRS4-AS1* promoter locus is also reflected in the UCSC ENCODE project database (see Supplementary Fig. S2)^32^. Additionally, we did not detect any changes in levels of the transcription factor CEBPbeta or the histone modifications H3K4me1 and H3K27ac at the enhancer locus, suggesting there was specific destabilization of RNA pol II and p300 at the enhancer locus following AS1eRNA depletion (Fig. 4C–E). Except for AS1eRNA-siRNA-2 reducing the level of H3K27ac, knockdown of AS1eRNA did not cause any changes in the levels of CEBPbeta, H3K4me1 and H3K27ac at the *DHRS4-AS1* promoter. As shown in our working model (Fig. 4F), enrichment of H3K4me1 and H3K27ac histone modifications, p300, RNA pol II and transcription factors such as CEBPbeta at the AS1 enhancer locus would provide the basal transcription machinery to transcribe AS1eRNA. With the involvement of RNA pol II and p300, AS1eRNA-driven DNA looping would bring the AS1 enhancer into close proximity with the *DHRS4-AS1* promoter. As a result, AS1eRNA-driven DNA looping could enhance the transcription of *DHRS4-AS1*.

### Activating histone modifications may lead to enhanced transcription of only *the DHRS4-AS1* NAT of the *DHRS4* gene cluster

**Figure 5:**
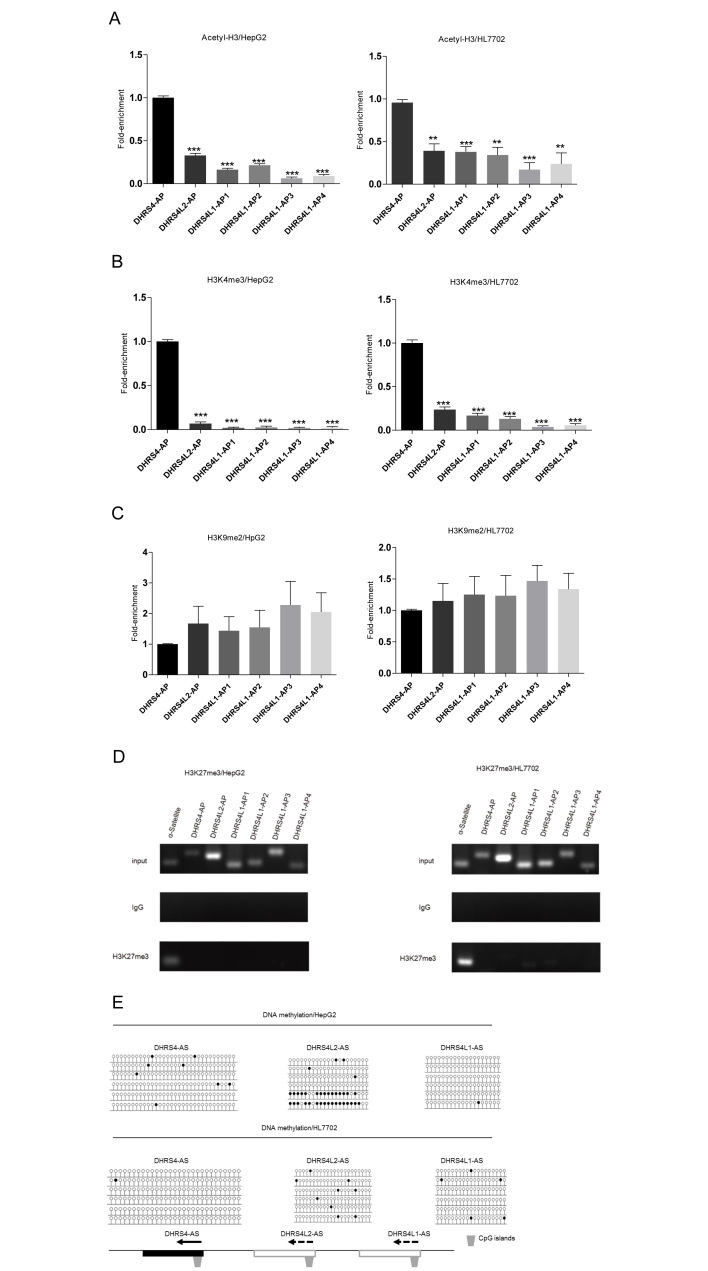
Activating histone modifications affect transcriptional activation for *DHRS4-AS1*, *DHRS4L2-AS* and *DHRS4L1-AS*. (**A–C**) Relative levels of acetyl-H3, H3K4me3 and H3K9me2 histone modifications were determined by ChIP analysis at predicted promoter regions within *DHRS4-AS1* and its putative homologous NATs of the *DHRS4* gene cluster in HepG2 cells and HL7702 cells. DHRS4-AP refers to the predicted promoter of *DHRS4-AS1*, and DHRS4L2-AP refers to the predicted promoter regions of putative NAT of *DHRS4L2*. Putative NAT promoter regions of *DHRS4L1* were termed DHRS4L1-AP1, DHRS4L1-AP2, DHRS4L1-AP3 and DHRS4L1-AP4 according to the position at different gene loci (Methods). An unpaired Student’s t test was used to evaluate the differences between DHSRS4-AP and the other promoters. Error bars represent the mean ± SEM of three independent experiments. *P < 0.05, **P < 0.01, ***P < 0.001. (**D**) H3K27me3 is present at very low levels at the predicted promoter regions within *DHRS4-AS1* and its putative homologous NATs in HL7702 and HepG2 cells. In ChIP assays, the PCR bands of α-satellite primers were used as the positive control to biologically validate the IP results. IgG was the negative control to ensure specificity of the ChIP reaction. (**E**) CpG island methylation status within *DHRS4-AS1* and its putative corresponding NAT regions was detected by using BSP analysis in HL7702 and HepG2 cells. Closed circles represent methylated CpG sites and open circles represent unmethylated CpG sites. The relative locations of CpG islands at the putative homologous NATs are shown (lower panel).

To investigate the influence of histone modifications on the transcription of *DHRS4-AS1* and its putative NATs of the *DHRS4* gene cluster, we performed ChIP assays to examine the distributions of acetyl-H3, H3K4me3, H3K9me2 and H3K27me3 in the predicted promoter regions of *DHRS4-AS1* and its corresponding putative NAT regions. As shown in Fig. 5A,B, the levels of acetyl-H3 and H3K4me3 were considerably greater in the *DHRS4-AS1* promoter than in the other promoter regions within the putative NATs for *DHRS4L2* and *DHRS4L1*, in both HL7702 and HepG2 cells. The distributions of H3K9me2 showed no statistical differences, whereas the levels of H3K27me3 were much lower in *DHRS4-AS1* (Fig. 5C,D). Characterization of the levels of DNA methylation in the CpG islands within *DHRS4-AS1* and its corresponding putative NAT regions demonstrated that they all displayed a hypomethylated status in both HepG2 and HL7702 cells (Fig. 5E). The results suggest that the activating acetyl-H3 and H3K4me3 histone modifications enhance transcription of *DHRS4-AS1* and may result in the expression of only the *DHRS4-AS1* NAT at the *DHRS4* gene cluster.

## Discussion

We previously demonstrated that the lncRNA *DHRS4-AS1* regulates all three *DHRS4* homologues of the *DHRS4* gene cluster in an epigenetic manner^6^. Here we extend those findings by showing that *DHRS4-AS1* expression is regulated by a novel downstream enhancer from which AS1eRNA is transcribed and which enhances *DHRS4-AS1* expression by mediating chromatin looping between the AS1 enhancer and the *DHRS4-AS1* promoter. In our case, the enhancing effect of AS1eRNA is on *DHRS4-AS1* lncRNA expression, which indirectly regulates *DHRS4* mRNA expression. Thus, our finding extends the function of eRNAs to include regulation of lncRNAs. Such an eRNA–lncRNA–mRNA network could be quite common, because eRNAs and lncRNAs constitute a large portion of the mammalian transcriptome.

Mounting evidence has revealed various enhancer mechanisms are at work under different conditions. For example, the androgen receptor (AR)-activated eRNA kallikrein-3e (KLK3e) participates in AR-driven DNA looping and mediates the interaction of this enhancer and the kallikrein-2 (*KLK2*) promoter to enhance *KLK2* gene expression^14^. In contrast, for the *Myod1* gene, eRNA expressed from the distal regulatory enhancer depends on chromatin accessibility, rather than chromatin looping between the enhancer and promoter to increase transcription^15^. The present study provides evidence that AS1eRNA participates in maintaining chromatin looping between the AS1 enhancer and the *DHRS4-AS1* promoter, which agrees with findings demonstrating that eRNAs can function as molecular bridges to mediate spatial interactions of distal enhancers and target promoters^14^,^33^. eRNAs are commonly found to be dynamically activated in response to specific stimuli such as hormones, and by signal transduction^14^,^24^,^34^. The natural endogenous expression of AS1eRNA may represent a novel function of enhancer transcripts, to fine-tune the level of gene expression without an exogenous stimulus.

Enhancer-like lncRNAs, uncovered as a class of lncRNAs, are spliced transcripts expressed from promoter-like regions (i.e. high H3K4me3, low H3K4me1), that show enhancer-like properties and are involved in long-range chromatin looping^35^,^36^,^37^. In contrast, eRNAs are produced from putative enhancer regions characterized by H3K27ac and high levels of H3K4me1 relative to H3K4me3, and are generally not spliced, as is our AS1 eRNA^38^,^39^. Although most eRNAs are non-polyadenylated bidirectional eRNAs^11^, a less common group of unidirectional polyadenylated transcripts is also identified from enhancer regions into which our AS1 eRNA falls^33^,^40^. The presence of RNA pol II and enhancer-associated marks such as H3K27ac and relatively high H3K4me1/H3K4me3 levels, have been widely used to predict enhancers expressing eRNAs, as we do^27^,^41^, whereas there are reports claim that enhancers expressing long ncRNAs that they have relatively high H3K4me3 level^42^,^43^. How large a fraction of lncRNAs including eRNAs or enhancer-like long ncRNAs are functional remains unknown and is subject to ongoing investigation^11^,^37^.

The mechanism that directs transcription of only the *DHRS4-AS1* NAT, but not putative NATs of *DHRS4L2* and *DHRS4L1*, aside from the regulatory enhancer for *DHRS4-AS1*, can be explained by the greater histone H3 acetylation (acetyl-H3) and histone H3 lysine 4 trimethylation (H3K4me3) activating modifications at the *DHRS4-AS1* promoter compared to at the corresponding duplicated regions of *DHRS4L2* and *DHRS4L1*. Whether DNA methylation plays a role in lncRNA expression is still deputed. Some reports have shown DNA methylation is associated with lncRNA expression, whereas other reports come to a different conclusion^44^,^45^. According to our results, DNA methylation does not seem play a role in lncRNA expression, consistent with other reports^45^,^46^.

Distal enhancers are a fundamental property of the mammalian regulatory genome, and almost half of all enhancers in each species appear to have evolved only recently^38^,^47^. The AS1 enhancer is specific to primates and changed rapidly during evolution along with the occurrence of the *DHRS4-AS1* exon 3 in primates. There was no detectable protein expression corresponding to either *DHRS4L2* or *DHRS4L1* in our tested cell lines. The silencing role of *DHRS4-AS1* on *DHRS4L2* and *DHRS4L1* could reflect an evolutionary strategy to silence the vast majority of newly duplicated genes in order to prevent potential deleterious genetic imbalances in organisms^48^,^49^. According to this model in the context of *DHRS4L2* and *DHRS4L1*, local transcription of homologous NATs to regulate themselves would be redundant and unnecessary. The enhancement effect of AS1eRNA on a single NAT, *DHRS4-AS1*, to regulate the entire *DHRS4* gene cluster would be an effective and economical way to coordinately manage gene expression. Our results elucidate the mechanism of the AS1 enhancer and epigenetic modifications on the enhancement of *DHRS4-AS1* expression and further help to explain the economic regulatory pattern at the tight *DHRS4* gene cluster.

## Methods

### Cell culture

The hepatocellular carcinoma HepG2 and normal hepatocyte HL7702 cell lines were obtained from the Cell Bank of the Chinese Academy of Sciences (Shanghai, China). Cells were grown RPMI 1640 medium containing 10% (v/v) fetal bovine serum at 37 °C in a 5% CO_2_ humidified incubator.

### RNA extraction, cDNA synthesis and real-time RT-PCR

Total RNA was extracted from cells following the manufacturer’s protocol (Trizol, Invitrogen). Primers for qPCR are listed in Supplementary Table S2. Quantifications were normalized to an internal control (β-actin).

### RNA interference

Transfection of HL7702 and HepG2 cells was performed with HiPerFect Transfection Reagents (QIAGEN) according to the manufacturer’s instructions. All siRNA oligonucleotides were designed by GenePharma (Shanghai, China). siRNA sequences are listed in see Supplementary Table S3.

### Dual-luciferase reporter assay

Predicted enhancers were amplified from genomic DNA and cloned in the forward or reverse orientation, upstream or downstream of the promoter into the pGL3-promoter vector (Promega), resulting in four constructs: [upstream/forward (U-F), upstream/reverse (U-R), downstream/forward (D-F), downstream/reverse (D-R)]. Putative enhancers were cloned into the *Xho*I (NEB) and *Hind*III-HF (NEB) sites upstream of the pGL3 promoter, or into the *Sal*I-HF (NEB) site downstream of the pGL3 promoter. All inserted sequences were verified by DNA sequencing. Luciferase activity was measured with the Dual-Luciferase Reporter Assay System (Promega) using a Glomax-multi detection system (Promega). The primers used here are listed in Supplementary Table S4.

### RACE

A SMARTer^®^ RACE 5′/3′ Kit (Clontech) was used to determine the 5′ end of AS1eRNA. The 3′ end of AS1eRNA was determined by using a 3′ RACE kit (TaKaRa). All RACE primers are listed in Supplementary Table S5.

### 3C assay

The 3C assay was performed as previously described but with some modifications^23^. Cross-linking was achieved by incubating 1 × 10^7^ cells in PBS containing 1% formaldehyde. Cell pellets were lysed and suspended in 1.2X restriction enzyme solution (*Hind*III-HF or *Eco*RI-HF) (400 units per 10^7^ cells). To stop the restriction digestion, 1.6% SDS (final concentration) was added, and samples were incubated at 65 °C for 25 min. 2000 units of T4 DNA ligase (NEB) per 10^7^ cells were added to perform DNA ligation at 16 °C for 4 h. The chromatin samples were reverse cross-linked with proteinase K (Invitrogen) at 65 °C overnight. 3C samples were then purified using phenol–chloroform extraction and amplified by qPCR analysis using the specific primers listed in Supplementary Table S6. All 3C PCR products were sequenced.

To normalize the efficiency of each primer set, 3C control PCR templates including all possible random ligations were generated with bacterial artificial chromosomes containing the *DHRS4-AS1*, *DHRS4* and *DHRS4L2* genes (CTD-3034B22), the *DHRS4L1* gene (CTD-2350C14), and the *ERCC3* gene (CTD-3251N23). To obtain standard curves covering the same range of 3C template concentrations, we made a series of dilutions for the control BAC clones. To correct for the ligation efficiency of different samples, the data were normalized to the *ERCC3* “control interaction frequencies”. All amplified PCR products were confirmed by sequencing.

### Bisulfite sequencing for DNA methylation analysis

We obtained the positions of CpG islands located in the antisense strands of the *DHRS4* gene cluster from the UCSC Genome Browser database (http://www.genome.ucsc.edu/).

Genomic DNA was bisulfite-modified using an Epitect Bisulfite Kit (QIAGEN). Primers were designed using MethPrimer (http://www.urogene.org/methprimer/index1.html) and Methyl Primer Express software (listed in Supplementary Table S7).

### Chromatin immunoprecipitation (ChIP) assay

We preformed ChIP assays with an EZ-Magna A kit (Millipore) according to the manufacturer’s protocol. The following antibodies were used for ChIP: anti-RNA pol II (05–623, Millipore), anti-acetyl histone H3 (17–408, Millipore), anti-H3K4me3 (17–614, Millipore), anti-H3K27me3 (17–622, Millipore), anti-H3K9me2 (17–648, Millipore), anti-H3K4me1 (ab8895, Abcam), anti-H3K27ac (ab4729, Abcam), anti-CEBPbeta(sc-150x, Santa Cruz) and anti-p300 (sc-585x, Santa Cruz). Immunoprecipitated DNA was analyzed by real-time PCR normalized with the input DNA. The putative promoters were predicted using PROSCAN version 1.7 (http://www-bimas.cit.nih.gov/molbio/proscan/). The predicted promoter regions located in the *DHRS4-AS1* gene spanned −976 to −726 nt relative to the TSS of *DHRS4-AS1*. In other contexts, the *DHRS4-AS1* predicted promoter region is located from nt 1405 to 1155 relative to the TSS of *DHRS4*. Relative to the TSS of *DHRS4L2*, the predicted promoter region of *DHRS4L2-AS* is located from nt 1490 to 1240 on the reverse strand. The homologous duplicated promoter region in the *DHRS4-AS1* region can be found in the antisense strand of *DHRS4L1*, but the homologous duplicated promoter region does not show promoter activity, termed AP1. There are another three promoter regions predicted for *DHRS4L1-AS* on the antisense strand: AP2, ranging from nt 1655 to 1905; AP3, ranging from nt 6042 to 6292; and AP4, ranging from nt 7299 to 7549 relative to the TSS of *DHS4L1*. Primers used here to perform qPCR are listed in Supplementary Table S8.

## Additional Information

**How to cite this article**: Yang, Y. *et al.* Enhancer RNA-driven looping enhances the transcription of the long noncoding RNA DHRS4-AS1, a controller of the *DHRS4* gene cluster. *Sci. Rep.* **6**, 20961; doi: 10.1038/srep20961 (2016).

## Supplementary Material

Supplementary Information
