# Supplementary material for: Enhancer RNA-driven looping enhances the transcription of the long noncoding RNA DHRS4-AS1, a controller of the DHRS4 gene cluster
Source: Sci Rep. 2016 Feb 11;6:20961. doi: 10.1038/srep20961 (PMC4750091; doi:10.1038/srep20961)
Supplement: Supplementary Information [file srep20961-s1.pdf]

Enhancer RNA-driven looping enhances the transcription of the long noncoding RNA DHRS4-AS1, a controller of the *DHRS4* gene cluster

Yingying Yang, Zhongjing Su, Xuhong Song, Bin Liang, Fanxing Zeng, Xiaolan Chang, Dongyang Huang*
Department of Cell Biology and Genetics and Key Laboratory of Molecular Biology in High Cancer Incidence Coastal Chaoshan Area of Guangdong Higher Education Institutes, Shantou University Medical College, Shantou 515041, China
*Correspondence to: Dongyang Huang; E-mail: huangdy@stu.edu.cn

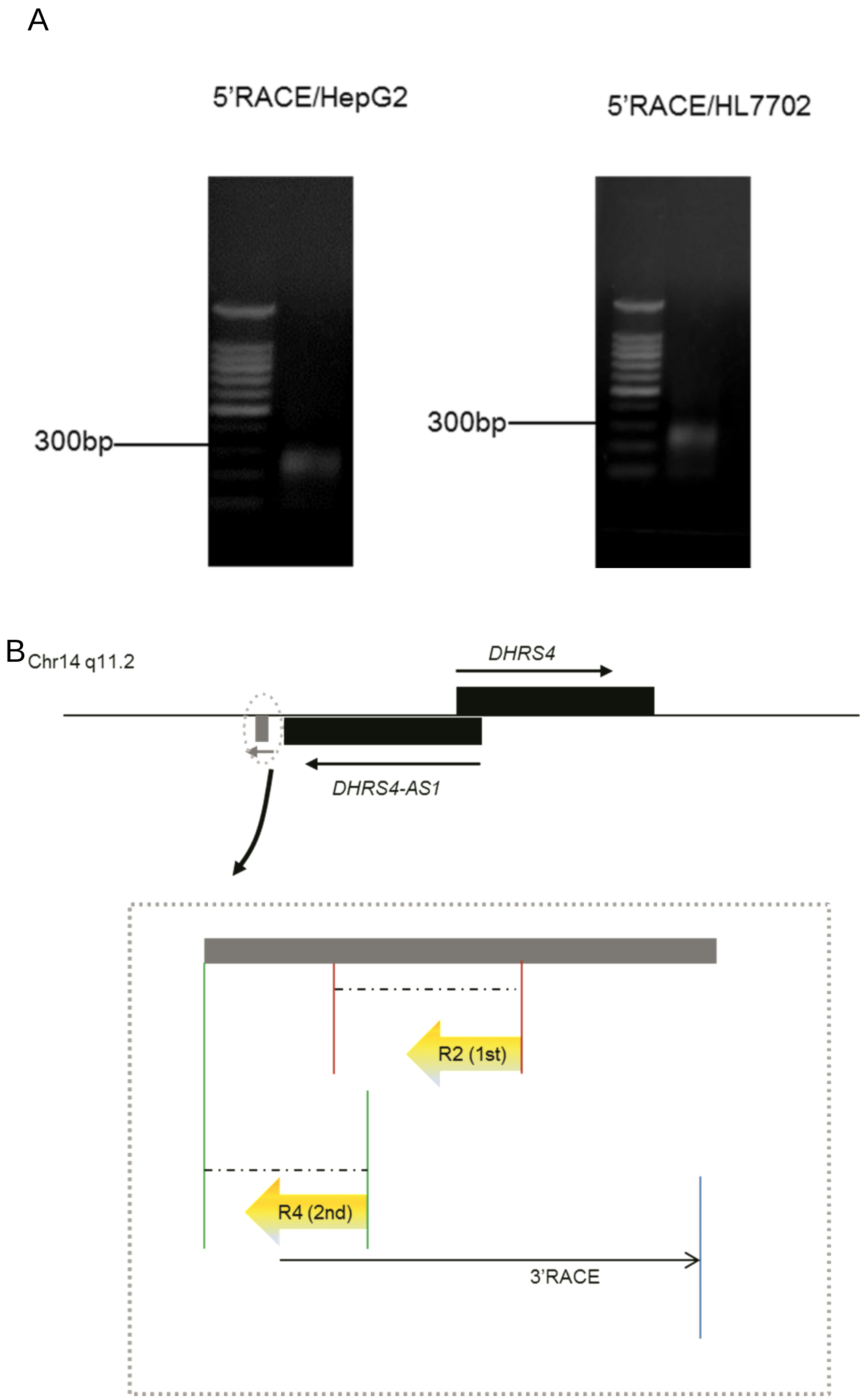
A
5’RACE/HepG2
5’RACE/HL7702
300bp
300bp
B
Chr14 q11.2
DHRS4
DHRS4-AS1
R2 (1st)
R4 (2nd)
3’RACE

C

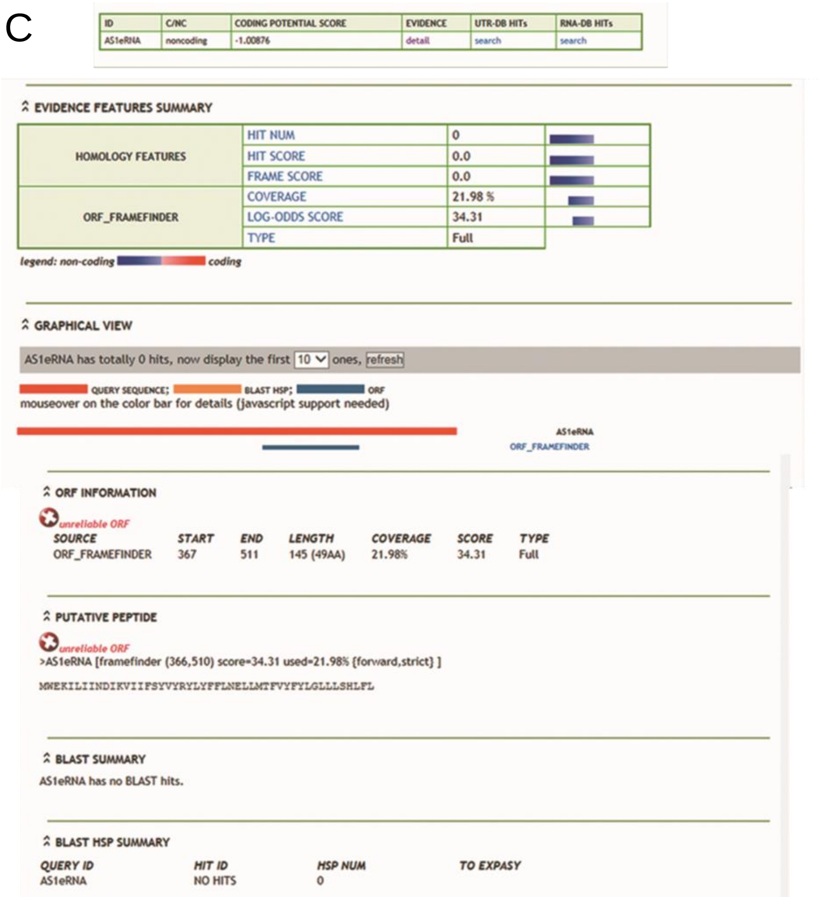

Figure S1. The AS1 enhancer produces eRNA. (A) 5' RACE was performed to determine the 5' end of AS1eRNA. (B) Schematic representation of the orientation of the primer extension used in the 5' RACE and 3' RACE shows the location of AS1eRNA relative to the *DHRS4-AS1* gene locus. R2 and R4 represent the 5' RACE outer and inner primers, respectively. The single-exon polyadenylated AS1eRNA was determined by RACE-PCR followed by DNA sequencing. (C) Snapshot from the Coding Potential Calculator (CPC) website is shown to discriminate AS1eRNA as "noncoding" with a coding potential score of -1.00876. Further evidence provided by CPC showed that there were no predicted protein functional domains, and no BLAST hits in the "unreliable ORF" of AS1eRNA.

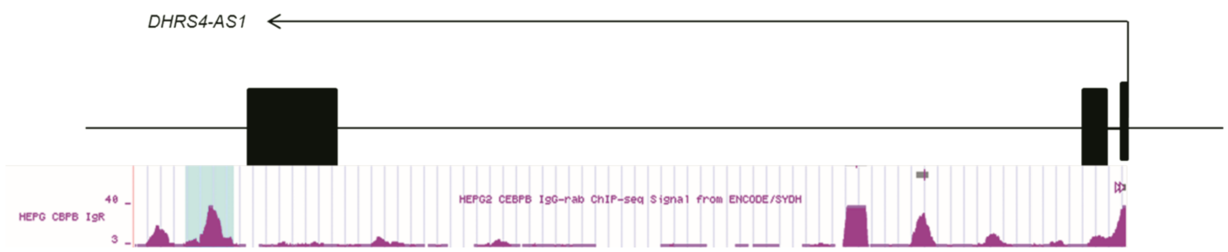

Figure S2: ENCODE tracks from the UCSC Genome Browser illustrate that the enrichment of CEBPbeta at the AS1 enhancer and the *DHRS4-AS1* promoter in HepG2 cells. The light blue rectangle represents the AS1 enhancer region.

Table S1: Homology comparisons between *DHRS4-AS1* and its corresponding putative NAT regions

Name	Overlapping DNA sequences	Predicted promoter regions
DHRS4-AS1 *vs* DHRS4L2-AS1	90%	98%
DHRS4-AS1 *vs* DHRS4L1-AS1	90%	91%
DHRS4L2-AS1 *vs* DHRS4L1-AS1	93%	92%

Table S2. Primers for qPCR

Name	Sequences
DHRS4- AS1-F	F: GCCTTGTGCATGGATCAGAC
DHRS4- AS1-R	R: GACCATCCCTGTGTGTTAGTGAG
AS1Erna-F	F: AAGGGCAAGGAGTGTGTCTTT
AS1eRNA-R	R: CACCATTACACAGGACTGCCT
β-actin-F	F: CTGGAACGGTGAAGGTGACA
β-actin-R	R: AAGGGACTTCCTGTAACAATGCA
c-MYC-F	F: CTTCTCTCCGTCCTCGGATTC
c-MYC-R	R: CAGAAGGTGATCCAGACTCTGACC

Table S3. SiRNA oligonucleotides

Name	Sequences
Sicontrol sense	UUCUCCGAACGUGUCACGUTT
Sicontrol antisense	ACGUGACACGUUCGGAGAATT
AS1eRNA-si1 sense	GUAGUACAUAGAGUCCCUGTT
AS1eRNA-si1 antisense	CAGGGACUCUAUGUACUACTT
AS1eRNA-si2 sense	CACUCAGCUUUAACAAACATT
AS1eRNA-si2 antisense	UGUUUGUUAAAGCUGAGUGTT

Table S4. Primers for dual-luciferase reporter assay

Primer name	Sequences
U-F-forward primer	CGACGCGTCGAGTACTATTGATCCAGAGGTGACC
U-F-reverse primer	GAAGATCTTAATCATGACACAAAACTCAGAAGC
U-R-forward primer	GAAGATCTGAGTACTATTGATCCAGAGGTGACC
U-R-reverse primer	CGACGCGTCTAATCATGACACAAAACTCAGAAGC
D-F/D-R-forward primer	ACGCGTCGACGAGTACTATTGATCCAGAGGTGACC
D-F/D-R-reverse primer	ACGCGTCGACTAATCATGACACAAAACTCAGAAGC
Random DNA-forward primer	ACGCGTCGACGCATAAGCAAAGGCCCTGAC
Random DNA-reverse primer	ACGCGTCGACTTCACCCTCACAGGATTGGC

Table S5. Primers for 3C array

Name	Sequences
*Hind*III-Anchor primer1	AATAGAAGAGTATGGCAGTTCAGAGG
*Hind*III-Anchor primer2	GGAGAGAGTATTTTTAGTTGGG
F1	GAGCTGTTTGTTCTGTGTTCTGATAG
F2	AGACTGAGAAGCAGTGCTGGTTC
F3	TCCATGTAAAGTGCTACCTTCAGG
F4	AGGAGTATGTTTGTGGTGTTCTCTG
F5	CTCTGTTTGCCTGGGTATCACTAG
ERCC3-F	CCCTACCCCATGTAGTGAATCC
ERCC3-R	ACTCCAAGGGTTATTTTGATGTTG
*Eco*RI-enhancer anchor	ACATCCCGAAGGTGTGATGC
*Eco*RI-L1-antisense promoter	GAAGCCGATCCTGTGAGCAG
*Hind*III-L2-antisense promoter	AGGATCTCATATCCCTCGC
Loading primer-F	CACTTCCCCAAGAAGAAAACGTG
Loading primer-R	CGGGCTGGTGAGTGATCAAAG

Table S6. Primers for RACE

Usage	Primer name	Sequences
5' RACE (1st round for nested PCR)	UPM	CTAATACGACTCACTATAGGGCAAGCAGTGG TATCAACGCAGAGT (long)
		CTAATACGACTCACTATAGGGC (short)
	AS1eRNA outer	GACTGCCTTTCAGGAAGGTTCTCTGC
5' RACE (2st round for nested PCR)	NUP	AAGCAGTGGTATCAACGCAGT
	AS1eRNA inner	GACACACTCCTTGCCCTTGTACTTCAAC
3' RACE	3' RACE outer primer	TACCGTCGTTCCACTAG
	AS1eRNA 3' RACE outer primer	CTCTAAGGCATGATGTTGCAGTAG

Table S7: Primers for BSP

Name	Sequences
DHRS4-AS-CGI-F	F: TGTAAGGTTTGAGGATAGGG
DHRS4-AS-CGI-R	R: AATTCCTACAAAAACCAAAAA
DHRS4L2-AS-CGI-F	F: TGGTTATTTGTATGGATTAGATTAG
DHRS4L2-AS-CGI-R	R: AAAAAACAACAACCAAAATCTAC
DHRS4L1-AS-CGI-F	F: TATTAGGGTTATTTTATTTGTGAG
DHRS4L1-AS-CGI-R	R: AAAAACAACAACCAAAAACTAC

Table S8. Primers for ChIP

Primer name	Sequences
DHRS4-AP-F	ACGACCACATGGGCCCC
DHRS4-AP-F	ACATAGTAGGCACACGTAGGGG
DHRS4L2-AP-F	GGAAGAGGTCGACAGCATGG
DHRS4L2-AP -R	GAACTAAGGCTGTGATCCAGGTC
L1-AP1-F	GACTACCACGTGGGCCCC
L1-AP1-R	CTTCAGAGCCCATGCTGTCC
L1-AP2-F	CCGATCCTGTGAGCAGAGAC
L1-AP2-R	CGTAGTAGAGACACATGGAGGTC
L1-AP3-F	TGTGTGGCATGTGGCAAAGAC
L1-AP3-R	CCCAGAAGGAAGTTTTTATCAACG
L1-AP4-F	CTCTCTTGCCATGTAACGCG
L1-AP4-R	GGTTTACACTTCTGCAGACTGCAG
AS1enhancer-F	AAGAAGGCTCTTTGTTGGGC
AS1enhancer-R	GGGATTGCTCAAGGTAGAAGG
